# Multiple Left Ventricular Ball Thrombi in a Patient With Nonischemic Cardiomyopathy Bridged to Heart-Lung Transplantation

**DOI:** 10.1016/j.jaccas.2025.105488

**Published:** 2025-09-18

**Authors:** Adam Zviman, Julie Zviman, Sarah Cipriano, Allen Burke, Manjula Ananthram

**Affiliations:** University of Maryland Medical Center, Baltimore, Maryland, USA

**Keywords:** cardiac transplant, chronic heart failure, thrombus

## Abstract

**Case Summary:**

We present a case of a young patient with a history of recurrent venous thromboembolism, stroke status post fibrinolysis, and titin-related dilated cardiomyopathy who developed cardiogenic shock in the setting of decompensated heart failure and concomitant large left ventricular thrombus comprising ∼75% of the cavity. Her shock was managed medically as she was evaluated for surgical heart failure therapies. The patient underwent a combined heart-lung transplant and was discharged after an uneventful postoperative course.

**Take-Home Messages:**

This case demonstrates that concomitant end-stage heart failure, large intracardiac thrombus, and severe pulmonary hypertension can be managed successfully with combined heart-lung transplantation.

## Graphical Abstract

**Figure:**
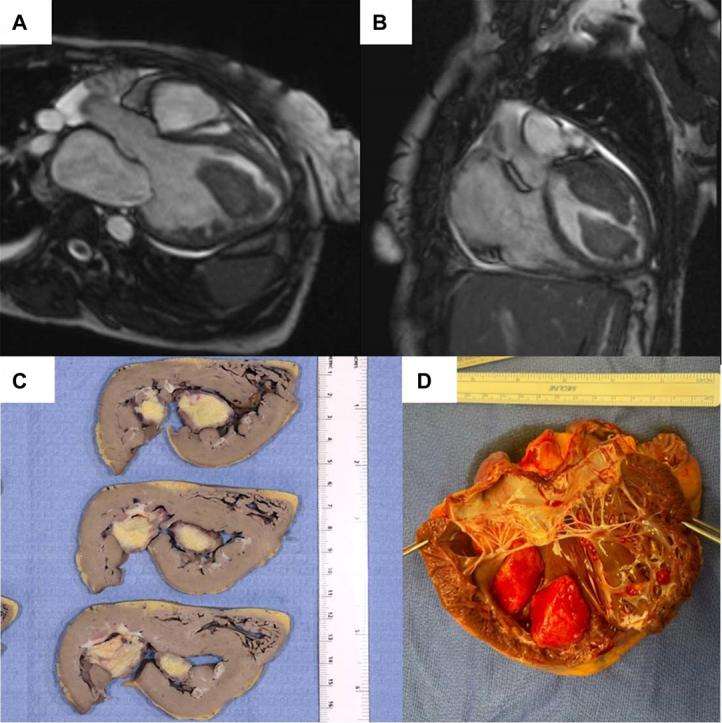

## Case

A 36-year-old woman with nonischemic dilated cardiomyopathy (DCM) due to a pathogenic titin (TTN) truncating variant presented in cardiogenic shock with progressive dyspnea and chest discomfort. Her history included prior submassive bilateral pulmonary emboli and an ischemic stroke 6 and 18 months prior, respectively. At the time of the stroke, transthoracic echocardiography and cardiac magnetic resonance imaging revealed de novo biventricular cardiomyopathy without evidence of intracardiac thrombus. The hypercoagulable workup was unremarkable, and the stroke was presumed to be cardioembolic. She was discharged on guideline-directed medical heart failure therapy with good neurologic and functional outcome. A primary prevention implantable cardioverter-defibrillator was not yet implanted.

**Figure 1:**
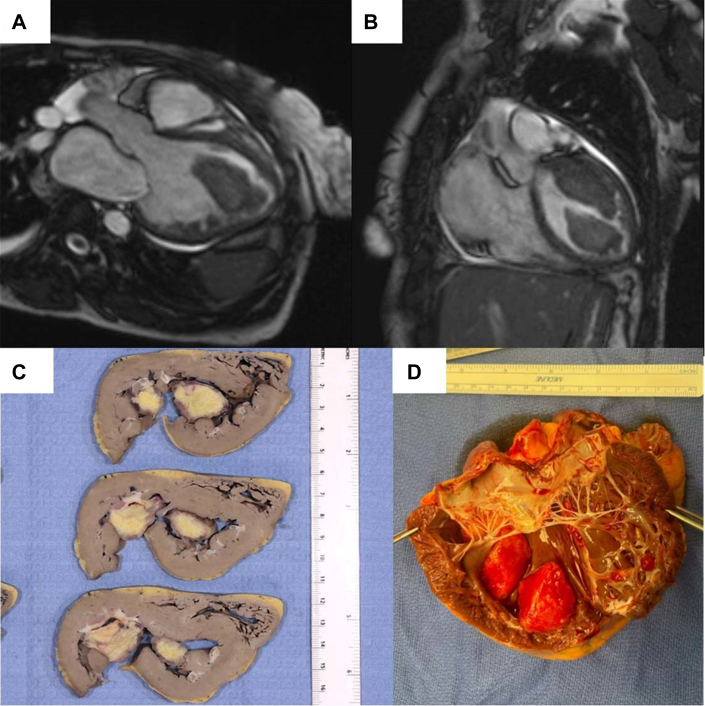
Cardiac Magnetic Resonance Images and the Anatomic Correlate After Native Heart Explant Representative cardiac magnetic resonance images showing left ventricular cavity dilation and large LV thrombus shown in long and short axis (A and B). (C and D) The anatomic correlate after native heart explant. Histopathologic examination shows the left ventricular thrombi have a bright yellow cut surface with <0.1-mm-thick white septations and focally hyperemic borders (not pictured).

On presentation, she was tachycardic and normotensive with signs of acute decompensated heart failure. Transthoracic echocardiography and cardiac magnetic resonance imaging were repeated (Figures 1A and 1B), revealing a severely dilated left ventricle (LV) (ejection fraction 11%) with a large multilobulated thrombus occupying ∼75% of the LV cavity (Video 1). Right-heart catheterization showed elevated biventricular filling pressures, combined precapillary and postcapillary pulmonary hypertension (pulmonary vascular resistance [PVR] 5.1 Wood Units), and low cardiac index despite dual inotrope therapy. Nitroprusside infusion was initiated with initial improvement in cardiac output but without significantly attenuated PVR.

Surgical thrombectomy was considered but deemed high risk due to poor LV function and embolization risk. left ventricular assist device placement was contraindicated due to thrombus burden and right ventricular failure. Isolated heart transplantation was relatively contraindicated due to severe, likely irreversible pulmonary hypertension from combined World Health Organization Group II and IV pathophysiology. After multidisciplinary evaluation, she was listed for combined heart-lung transplantation as United Network for Organ Sharing status 1 by exception.

She underwent successful en-bloc heart-lung transplantation on hospital day 48, with native heart explant demonstrating the large thrombus (Figures 1C and 1D). Her postoperative course was uncomplicated; she was discharged on low-molecular-weight heparin and later transitioned to apixaban. At 1-year follow-up, she reports excellent functional status with normal graft function and no recurrent thrombi.

This case underscores several key principles in the management of end-stage heart failure complicated by massive LV thrombus. TTN-related DCM can rapidly progress and lead to thrombus formation via low-flow states, despite not being intrinsically thrombogenic. In rare, high-risk cases where thrombectomy and left ventricular assist device are not feasible, and pulmonary hypertension contraindicates isolated heart transplantation, heart-lung transplantation may be the only therapeutic option.

International Society for Heart and Lung Transplantation guidelines consider PVR >5 WU a relative contraindication to heart-only transplantation given the risk of postoperative right heart failure. Heart-lung transplantation, though rare, is a lifesaving intervention for select patients with biventricular failure and irreversible pulmonary vascular disease. Our case represents a rare use of this approach in TTN-related cardiomyopathy complicated by massive intracardiac thrombus.

Finally, this case highlights the importance of genetic evaluation in DCM. Identification of a TTN mutation facilitated appropriate family counseling and cascade screening, which is increasingly recognized as critical in improving early detection and management of genetic cardiomyopathies.

## Funding Support and Author Disclosures

The authors have reported that they have no relationships relevant to the contents of this paper to disclose.

## Appendix

Video 1 A Case of Multiple Left Ventricular Ball Thrombi in a Patient With Nonischemic Cardiomyopathy Bridged to Heart-Lung Transplantation

